# Associations of seated dual-task ergometer training with cognitive and mobility outcomes in older adults with disabling conditions undergoing inpatient convalescent rehabilitation: a retrospective observational study

**DOI:** 10.1080/07853890.2026.2732560

**Published:** 2026-10-03

**Authors:** Makoto Hasui, Shunji Takashima, Machiko Suzuki, Takayuki Isobe, Yutaro Yamashita, Yohei Kurotobi, Katsuya Yamauchi

**Affiliations:** a Department of Rehabilitation Medicine, JA Shizuoka Koseiren Enshu Hospital, Shizuoka, Japan; b Department of Rehabilitation Medicine, Hamamatsu University School of Medicine, Shizuoka, Japan

**Keywords:** Dual-task, ergometer training, cognition, gait, convalescent rehabilitation, inpatients

## Abstract

**Objective:**

To examine associations between a 4-week seated dual-task (DT) ergometer program and cognitive and mobility outcomes in older convalescent rehabilitation inpatients.

**Materials and methods:**

This retrospective observational study included 125 rehabilitation episodes involving older adults undergoing inpatient convalescent rehabilitation , primarily for orthopaedic disorders and stroke. Participants received seated DT ergometer training (*n* = 79) or conventional recumbent ergometer exercise (*n* = 46) for 4 weeks. Primary outcomes were the Mini-Mental State Examination (MMSE), Trail Making Test–Japanese Part A (TMT-J Part A), 10-m DT walking time, and the Timed Up and Go test (TUG). Analysis of covariance models adjusted post-training outcomes for baseline values, age, and diagnosis using HC3 robust standard errors.

**Results:**

Adjusted between-group differences (DT minus conventional exercise) were nonsignificant for MMSE (0.08 points; 95% confidence interval [CI], −0.78 to 0.93; *p* = 0.862), TMT-J Part A (−6.59 s; 95% CI, −22.06 to 8.87; *p* = 0.403), DT walking time (−0.89 s; 95% CI, −2.77 to 0.99; *p* = 0.354), and TUG (−0.97 s; 95% CI, −2.62 to 0.67; *p* = 0.246). The six-sessions-per-week sensitivity analysis yielded consistent results. Exploratory analyses showed greater improvements in DT walking time (*p* = 0.003) and TUG (*p* = 0.016) in the MMSE ≥27 stratum. Within standard TMT-J limits, adjusted Part A time was shorter in the DT group (*p* = 0.035), whereas the unadjusted change-score comparison was nonsignificant (*p* = 0.182).

**Conclusions:**

Seated DT ergometer training did not demonstrate a clear advantage over conventional ergometer exercise in cognitive or mobility outcomes. Exploratory findings are hypothesis-generating. Prospective randomized studies with standardized training exposure are needed.

## KEY MESSAGES

- Seated dual-task ergometer training was not associated with significantly better cognitive or mobility outcomes than conventional ergometer exercise after adjustment.
- Exploratory analyses suggested potential mobility improvements among patients with relatively preserved cognition and possible sensitivity of TMT-J Part A under standard completion limits.
- Prospective randomized studies with standardized training exposure are needed to determine the specific effects of seated dual-task ergometer training.

## Introduction

Population ageing has led to an increasing number of older adults requiring rehabilitation after acute illness or injury. Older inpatients undergoing rehabilitation commonly experience concurrent declines in physical and cognitive function, which contribute to impaired mobility, loss of independence, and an increased risk of falls.

Walking and other mobility-related activities require the integration of motor control with attention, executive function, and information processing [1]. Dual-task (DT) paradigms are therefore widely used to assess cognitive–motor interference during movement [2]. DT performance is frequently impaired with ageing and neurological disease, and greater DT interference has been associated with gait instability, functional decline, and falls [2,3].

DT training has been proposed as a rehabilitation strategy for improving cognitive and motor performance. Systematic reviews and meta-analyses have reported improvements in cognition, gait, and balance after DT training in older adults and individuals with neurological or cognitive impairment [4–6]. However, the findings have been heterogeneous, and whether DT training provides additional benefits beyond physical exercise alone remains uncertain [5,7].

Most previous DT interventions have involved standing or walking tasks [4,5]. Although these approaches are directly relevant to daily mobility, they may be difficult to implement in patients with impaired balance, limited walking ability, fatigue, or medical restrictions. Seated cycle ergometry provides repetitive lower-limb and aerobic exercise with lower balance demands and can be adapted for patients with restricted ambulatory function. Combining cognitive tasks with seated cycling may therefore provide a practical means of introducing cognitive–motor training before walking-based DT practice becomes feasible.

Evidence regarding cycling-based DT exercise remains limited and inconsistent in convalescent rehabilitation populations. These populations predominantly comprise older patients with heterogeneous diagnoses and wide variations in cognitive and physical function, including individuals for whom walking-based DT training may not yet be practical [7].

The purpose of this retrospective observational study was to examine the associations between a 4-week seated DT ergometer training program and cognitive and mobility outcomes in inpatients undergoing convalescent rehabilitation. We hypothesized that seated DT ergometer training would be associated with greater improvements in cognitive and mobility outcomes than conventional ergometer training alone.

## Materials and methods

### Study design

This retrospective observational study was conducted in the convalescent rehabilitation ward of JA Shizuoka Koseiren Enshu Hospital, Shizuoka, Japan, between May 2016 and April 2025. Clinical data were retrospectively extracted from the electronic medical records of consecutive eligible inpatients admitted during the study period. Outcome measures were assessed immediately before and after the 4-week training period as part of routine clinical practice using assessment procedures implemented in the rehabilitation ward.

### Participants

The cohort comprised older inpatients with disability arising from a range of primary diagnoses: orthopedic disorders in 79 rehabilitation episodes (63.2%), stroke in 43 (34.4%), and deconditioning in 3 (2.4%). At baseline, mean MMSE scores were approximately 24 in both groups, indicating that the cohort included patients with cognitive impairment. Mean TUG time was 21.3 s in both groups, demonstrating limited functional mobility. Detailed baseline characteristics are presented in Table 1.

**Table 1.** Baseline characteristics of the study cohort.

Variable	DT group (*n* = 79)	Conventional ergometer group (*n* = 46)	*P* value
Age, years	77.8 ± 9.1	81.2 ± 9.8	0.015
Male sex, n (%)	27 (34.2%)	16 (34.8%)	1.000
Diagnosis, n (%)			0.002
Orthopedic disorder	41 (51.9%)	38 (82.6%)	
Stroke	36 (45.6%)	7 (15.2%)	
Deconditioning	2 (2.5%)	1 (2.2%)	
Scheduled training frequency, n (%)			<0.001
3 sessions/week	22 (27.8%)	0 (0%)	
6 sessions/week	57 (72.2%)	46 (100%)	
MMSE	24.0 ± 4.0	24.2 ± 3.2	0.835
TMT-J Part A, s	151.6 ± 66.4 (*n* = 75)	144.8 ± 44.5 (*n* = 45)	0.989
TMT-J Part B, s	235.7 ± 124.1 (*n* = 65)	267.2 ± 225.8 (*n* = 31)	0.638
10-m comfortable walking time, s	18.0 ± 11.6	17.0 ± 7.2	0.699
10-m maximal walking time, s	14.5 ± 9.2	13.0 ± 5.7	0.828
DT walking time, s	21.0 ± 12.6	20.4 ± 8.9 (*n* = 43)	0.614
TUG, s	21.3 ± 13.1 (*n* = 77)	21.3 ± 12.5 (*n* = 44)	0.649

Data are presented as mean ± standard deviation or number (percentage), as appropriate. The number of available observations is shown for variables with missing data. Continuous variables were compared using the Mann–Whitney U test. Categorical variables were compared using the chi-square test or Fisher’s exact test, as appropriate. Analyses were based on available cases, and missing values were not imputed.

Abbreviations: DT, dual-task; MMSE, Mini-Mental State Examination; TMT-J, Trail Making Test–Japanese; TUG, Timed Up and Go test.

Patients were eligible if they completed either seated ergometer-based DT training using the CB01 device or conventional recumbent ergometer exercise for four consecutive weeks and had baseline and post-training data available for at least one corresponding outcome measure.

A total of 125 rehabilitation episodes were included in the analysis, comprising 79 in the DT group and 46 in the conventional ergometer group. One patient was admitted twice during the study period. Because the two admissions represented separate rehabilitation episodes, both episodes were treated as independent observations.

Training allocation was not randomized and reflected routine clinical practice in the rehabilitation ward. Selection of the training modality was influenced by clinical and operational factors, including availability of the CB01 device, the number of patients using the equipment, rehabilitation scheduling, and therapist judgment regarding the patient’s ability to safely perform simultaneous cognitive and motor tasks. Consequently, baseline differences between the groups were anticipated and were addressed in the adjusted analyses.

The study analyzed routinely collected data obtained during standard rehabilitation care and did not introduce any additional intervention beyond usual clinical practice. Outcome assessors were not prospectively blinded to training allocation, and assessor awareness of group allocation could not be reliably determined from the available records.

The study protocol was approved by the Institutional Review Board of JA Shizuoka Koseiren Enshu Hospital (Approval No. 2025-05-01; approved May 13, 2025). The study was conducted in accordance with the Declaration of Helsinki. The requirement for written informed consent was waived because of the retrospective design and use of anonymized clinical data. An opt-out procedure was implemented, and study information was publicly disclosed to provide patients with an opportunity to decline participation.

This study was reported in accordance with the Strengthening the Reporting of Observational Studies in Epidemiology statement.

### Interventions

Participants in the DT group performed seated DT ergometer training using the CB01 device (Inter Reha Co., Ltd., Japan) for four consecutive weeks. Training was scheduled either three or six times per week according to routine rehabilitation operations, including equipment availability, the number of patients using the device, and rehabilitation scheduling.

Each session consisted of approximately 13 min of cycling while simultaneously performing six standardized touchscreen-based cognitive tasks: number selection, rock–paper–scissors, digit span, color matching, animal matching, and simple arithmetic. Task difficulty was automatically adjusted according to individual performance. A touch pen was permitted for participants with upper-limb impairment. Exercise intensity was targeted at a rating of 3, corresponding to ‘moderate,’ on the modified Borg scale.

Participants in the conventional ergometer group performed recumbent ergometer cycling without concurrent cognitive tasks for the same approximate session duration and target exercise intensity. All participants in the conventional ergometer group were scheduled to receive six sessions per week throughout the 4-week period.

The available records indicated the scheduled training frequency, but session-level documentation, including session duration, varied by treating therapist. Because absence of a documented duration could not be distinguished from absence of training, the actual number of sessions completed by each participant and cumulative training dose could not be determined reliably. Consequently, adherence and dose–response relationships could not be evaluated.

### Outcome measures

The primary outcome measures comprised two cognitive measures (MMSE and TMT-J Part A) and two performance-based mobility measures (10-m DT walking time and TUG).

### Cognitive outcomes

Cognitive outcomes included the Mini-Mental State Examination (MMSE), the Trail Making Test–Japanese (TMT-J) Part A and Part B [8], and ΔTMT, calculated as the TMT-J Part B completion time minus the Part A completion time.

The MMSE is a 30-point screening measure of global cognitive function, including orientation, registration, attention, calculation, recall, language, and visuospatial ability. Higher scores indicate better global cognitive performance [9].

TMT-J Part A primarily assesses visual scanning, sustained attention, and processing speed. Participants are required to connect numbered targets in ascending order, and a shorter completion time indicates better performance [8,10].

TMT-J Part B additionally requires set shifting and cognitive flexibility by asking participants to alternate between numerical and Japanese syllabic sequences. A shorter completion time indicates better performance [8,10]. ΔTMT was used as an exploratory index of the additional executive demand of Part B relative to Part A; a larger value indicates a greater increase in completion time associated with the set-shifting component.

For the primary analyses, all recorded TMT-J completion times were retained, including values exceeding the standard discontinuation limits, because these values represented completed assessments recorded in routine clinical practice. Source-record review showed that neither Part A nor Part B was administered at six assessment occasions across five rehabilitation episodes: at both assessment points in one episode, at baseline only in one, and at post-training only in three. For other missing TMT-J values, testing had been attempted but could not be completed; specific reasons for noncompletion were not documented. Because discontinuation procedures were not consistently applied in routine clinical practice, some participants had recorded completion times exceeding the standard limits [8].

### Mobility outcomes

Mobility outcomes included 10-m comfortable walking time, 10-m maximal walking time, 10-m DT walking time, DT gait cost, and the Timed Up and Go test (TUG).

For the 10-m comfortable walking test, participants were instructed to walk at their usual comfortable pace. For the 10-m maximal walking test, they were instructed to walk as quickly and safely as possible. During the walking assessments, participants used the walking aid judged by the rehabilitation therapist to be necessary for safe performance. Walking-aid use at baseline and post-training was confirmed from the medical records for all rehabilitation episodes. Shorter walking times indicate better walking performance.

For the 10-m DT walking test, participants walked while performing serial subtraction by twos. Serial subtraction by twos was selected as a cognitive task that could be applied to rehabilitation inpatients with a broad range of cognitive abilities. A shorter DT walking time indicates better mobility under concurrent cognitive demand.

DT gait cost was calculated as follows:

$$\text{DT gait cost }\left(\%\right)=\left[\left(\text{DT walking time}-\text{ST walking time}\right)/\left(\left(\text{DT walking time}+\text{ST walking time}\right)/2\right)\right]\;\times \;100.$$

A larger positive value indicates greater proportional slowing under the DT condition and, therefore, greater cognitive–motor interference [11].

The TUG assesses functional mobility and dynamic balance [12]. Participants were instructed to stand from a chair, walk 3 m, turn, return to the chair, and sit down. A shorter completion time indicates better functional mobility.

All outcome measures were assessed immediately before and after the 4-week training period. At each assessment time point, all outcomes were administered in a single session by rehabilitation therapists. The MMSE was interviewer-administered, the TMT-J was administered using standard paper-and-pencil forms, and the walking tests and TUG were timed using a stopwatch. Missing mobility outcome data were treated as missing. Whether an assessment had not been performed or the result had not been entered into the dataset could not be determined retrospectively. The participant selection process is illustrated in Figure 1.

**Figure 1.**
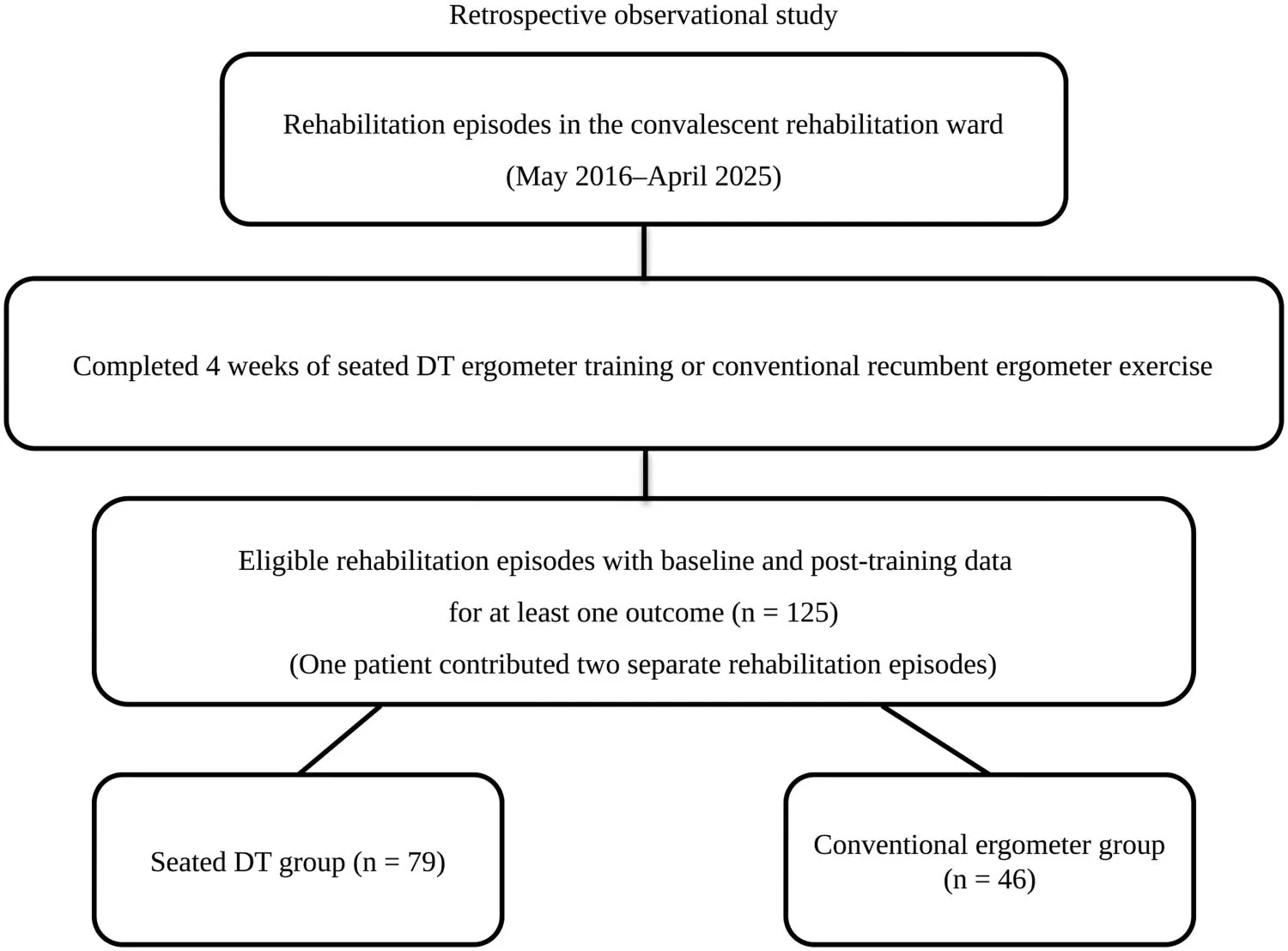
Flowchart of study cohort selection. Rehabilitation episodes occurring in the convalescent rehabilitation ward between May 2016 and April 2025 were retrospectively reviewed. Episodes were included when patients completed 4 weeks of either seated dual-task (DT) ergometer training or conventional recumbent ergometer exercise and had baseline and post-training data available for at least one corresponding outcome. A total of 125 rehabilitation episodes were included: 79 in the DT group and 46 in the conventional ergometer group. One patient contributed two separate rehabilitation episodes.

### Statistical analysis

Continuous variables are presented as mean ± standard deviation, whereas categorical variables are presented as frequencies and percentages. Distributional assumptions were evaluated using the Shapiro–Wilk test together with visual inspection of histograms and Q–Q plots.

Baseline continuous variables were compared between the groups using the Mann–Whitney U test. Categorical variables were compared using the χ^2^ test or Fisher’s exact test, as appropriate.

Within-group changes from baseline to post-training were assessed using paired t-tests when the change scores were approximately normally distributed and Wilcoxon signed-rank tests otherwise. Between-group differences in change scores were evaluated using the Mann–Whitney U test. Effect sizes for between-group Mann–Whitney U tests were calculated as r = |Z|/√N, where N was the total number of observations included in each comparison.

For the primary outcomes—MMSE, TMT-J Part A, DT walking time, and TUG—analysis of covariance (ANCOVA) was performed using the post-training value as the dependent variable and training group as the fixed factor. The corresponding baseline value, age, and diagnosis were included as covariates. Diagnosis was entered as a categorical variable. Adjusted group differences and 95% confidence intervals (CIs) were estimated using heteroscedasticity-consistent HC3 robust standard errors because heteroscedasticity was observed for some outcomes.

To examine the potential influence of scheduled training frequency, the ANCOVA models were repeated in a sensitivity analysis restricted to participants scheduled to receive six training sessions per week. A post hoc sensitivity analysis was also performed for the primary mobility outcomes by repeating the ANCOVA models for DT walking time and TUG after excluding the nine rehabilitation episodes in which walking-aid use changed between baseline and post-training assessments.

An additional exploratory sensitivity analysis was performed for the TMT-J outcomes. The analyses were repeated after excluding recorded completion times exceeding the standard discontinuation limits of 180 s for Part A and 300 s for Part B [8]. For ΔTMT, participants were included only when both Part A and Part B values were within the corresponding standard limits at both assessment time points.

Participants were further stratified according to baseline MMSE scores into low (≤21), intermediate (22–26), and preserved (≥27) cognitive-function groups. Exploratory subgroup analyses compared change scores between the training groups within each stratum using the Mann–Whitney U test. The TMT-J sensitivity analyses and MMSE-stratified subgroup analyses were considered hypothesis-generating and were not adjusted for multiple comparisons.

All analyses were conducted using available cases for each outcome, and missing values were not imputed. Consequently, the number of observations included varied across outcome analyses. Because this was a retrospective study using an available clinical cohort, no a priori sample size calculation was performed. Nonsignificant findings were therefore not interpreted as demonstrating equivalence or absence of an association.

Statistical significance was defined as a two-sided P value <0.05. All analyses were performed using Python version 3.11 (Python Software Foundation, Wilmington, DE, USA).

## Results

### Participant characteristics

Baseline demographic and clinical characteristics are summarized in Table 1. A total of 125 rehabilitation episodes were included in the analysis: 79 in the DT group and 46 in the conventional ergometer group. Analyses were performed using available cases for each outcome without imputation of missing values; therefore, the number of observations varied across analyses.

The DT group was significantly younger than the conventional ergometer group (77.8 ± 9.1 vs. 81.2 ± 9.8 years, *p* = 0.015), and the distribution of diagnostic categories differed significantly between the groups (*p* = 0.002). No statistically significant between-group differences were observed in baseline cognitive or mobility outcomes (all *p* > 0.05). Age, diagnosis, and the baseline value of each outcome were included as covariates in the adjusted analyses.

### Cognitive outcomes

Changes in the primary cognitive outcomes are summarized in Table 2, and secondary cognitive outcomes are presented in Supplementary Table S1. Paired pre- and post-training data were available for 120 observations for the MMSE, 115 for TMT-J Part A, and 88 for TMT-J Part B and ΔTMT.

**Table 2.** Primary cognitive and mobility outcomes.

Outcome	DT group, n	DT group Δ (95% CI)	Conventional ergometer group, n	Conventional ergometer group Δ (95% CI)	Between-group P	Effect size, r	Adjusted difference, DT − conventional ergometer (95% CI)	Adjusted *P*
MMSE	74	1.20 (0.51 to 1.89)	46	0.63 (−0.17 to 1.43)	0.480	0.065	0.08 (−0.78 to 0.93)	0.862
TMT-J Part A (s)	74	−17.57 (−28.78 to −6.35)	41	−13.16 (−27.27 to 0.95)	0.293	0.098	−6.59 (−22.06 to 8.87)	0.403
DT walking time (s)	76	−4.36 (−5.64 to −3.09)	41	−3.34 (−5.21 to −1.47)	0.123	0.143	−0.89 (−2.77 to 0.99)	0.354
TUG (s)	76	−5.12 (−6.43 to −3.81)	42	−4.39 (−6.38 to −2.41)	0.271	0.101	−0.97 (−2.62 to 0.67)	0.246

Values are presented as mean changes from baseline with 95% CIs. Negative change values indicate shorter completion times and therefore improvement for the time-based outcomes. Between-group P values for change scores were obtained using the Mann–Whitney U test. Effect sizes were calculated as the absolute value of the standardized Z statistic divided by the square root of the total sample size for each comparison. Adjusted differences represent the estimated between-group differences in post-training values from ANCOVA models, with the DT group minus the conventional ergometer group reported. Models were adjusted for the corresponding baseline value, age, and diagnosis, with diagnosis entered as a categorical variable. Heteroscedasticity-consistent HC3 robust standard errors were used. Analyses were based on available cases, and missing values were not imputed.

Abbreviations: ANCOVA, analysis of covariance; CI, confidence interval; DT, dual-task; MMSE, Mini-Mental State Examination; TMT-J, Trail Making Test–Japanese; TUG, Timed Up and Go test.

Between-group comparisons of change scores showed no statistically significant differences in the MMSE, TMT-J Part A, TMT-J Part B, or ΔTMT (all *p* > 0.05). After adjustment for the corresponding baseline value, age, and diagnosis, no significant between-group differences were observed in the MMSE (adjusted mean difference, 0.08 points; 95% CI, −0.78 to 0.93; *p* = 0.862) or TMT-J Part A (adjusted mean difference, −6.59 s; 95% CI, −22.06 to 8.87; *p* = 0.403).

Within the DT group, the MMSE and TMT-J Part A improved significantly over the 4-week period. TMT-J Part B did not change significantly (*p* = 0.087), whereas ΔTMT also showed no significant change. No significant within-group changes in cognitive outcomes were observed in the conventional ergometer group. These within-group findings were not accompanied by significant between-group differences in change scores.

As an exploratory sensitivity analysis, the TMT-J analyses were repeated after excluding recorded completion times exceeding the standard discontinuation limits of 180 s for Part A and 300 s for Part B. Paired TMT-J Part A data within the standard limit were available for 76 observations, including 48 in the DT group and 28 in the conventional ergometer group. The adjusted post-training TMT-J Part A completion time was significantly shorter in the DT group than in the conventional ergometer group (adjusted mean difference, −15.86 s; 95% CI, −30.64 to −1.09; *p* = 0.035). However, the unadjusted between-group comparison of change scores remained nonsignificant (*p* = 0.182). No significant between-group differences were observed for TMT-J Part B or ΔTMT in the corresponding sensitivity analyses (Supplementary Table S3).

### Gait and mobility outcomes

Changes in the primary mobility outcomes are summarized in Table 2, and secondary mobility outcomes are presented in Supplementary Table S1. Paired pre- and post-training data were available for 124 observations for comfortable walking time, 117 for DT walking time, 123 for maximal walking time, and 118 for the TUG. Walking-aid use remained unchanged in 116 rehabilitation episodes and changed in 9: 4 in the DT group and 5 in the conventional ergometer group. The documented changes were from a walker to walking without an aid in four episodes, from a walker to a cane in two episodes, and from a cane to walking without an aid in three episodes.

Both groups demonstrated significant within-group improvements in comfortable walking time, maximal walking time, DT walking time, and TUG over the intervention period (all within-group *p* < 0.001). However, between-group comparisons of change scores showed no statistically significant differences in any gait or mobility outcome.

Adjusted ANCOVA analyses similarly showed no significant between-group differences in DT walking time (adjusted mean difference, −0.89 s; 95% CI, −2.77 to 0.99; *p* = 0.354) or TUG (adjusted mean difference, −0.97 s; 95% CI, −2.62 to 0.67; *p* = 0.246) after adjustment for the corresponding baseline value, age, and diagnosis. In the post hoc sensitivity analysis excluding the nine episodes with changes in walking-aid use, adjusted between-group differences remained nonsignificant for DT walking time (−1.15 s; 95% CI, −3.04 to 0.75; *p* = 0.236) and TUG (−1.30 s; 95% CI, −3.00 to 0.40; *p* = 0.135) (Supplementary Table S4).

Sensitivity analyses restricted to observations from patients scheduled to receive six training sessions per week yielded findings consistent with the primary analyses, with no statistically significant adjusted between-group differences in the MMSE, TMT-J Part A, DT walking time, or TUG (Supplementary Table S2).

### Exploratory subgroup analyses

Exploratory subgroup analyses stratified according to baseline MMSE score (≤21, 22–26, and ≥27) are summarized in Table 3.

**Table 3.** Exploratory subgroup analyses of mobility outcomes according to baseline MMSE score.

Outcome	Baseline MMSE subgroup	DT group, n	DT group Δ, mean ± SD	Conventional ergometer group, n	Conventional ergometer group Δ, mean ± SD	Between-group *P*	Effect size, r
DT walking time (s)	≤21	19	−3.23 ± 5.67	7	−1.59 ± 4.88	0.544	0.119
	22–26	38	−4.08 ± 4.00	22	−5.65 ± 6.63	0.634	0.061
	≥27	19	−6.06 ± 7.78	12	−0.13 ± 2.69	0.003	0.543
TUG (s)	≤21	19	−4.41 ± 4.00	7	−6.10 ± 10.78	0.452	0.147
	22–26	38	−4.74 ± 6.11	24	−5.18 ± 5.63	0.608	0.065
	≥27	19	−6.59 ± 6.42	11	−1.58 ± 3.38	0.016	0.440

Data are presented as mean changes from baseline ± standard deviation. Negative values indicate shorter completion times after training and therefore improvement. Between-group comparisons of change scores were performed using the Mann–Whitney U test. Effect sizes were calculated as the absolute value of the standardized Z statistic divided by the square root of the total sample size for each comparison. These subgroup analyses were exploratory and were not adjusted for multiple comparisons.

Abbreviations: DT, dual-task; MMSE, Mini-Mental State Examination; SD, standard deviation; TUG, Timed Up and Go test.

Among patients with baseline MMSE scores ≥27, the DT group showed greater improvements in DT walking time (*p* = 0.003) and TUG (*p* = 0.016) than the conventional ergometer group. No statistically significant between-group differences were observed in the intermediate- or low-MMSE subgroups.

Because these analyses were exploratory, involved relatively small subgroup sample sizes, and were not adjusted for multiple comparisons, the findings should be interpreted cautiously and regarded as hypothesis-generating.

## Discussion

This study examined the associations between a 4-week seated DT ergometer training program and cognitive and mobility outcomes in a heterogeneous, predominantly older inpatient population undergoing convalescent rehabilitation. In the primary analyses, no clear between-group differences were observed in cognitive or mobility outcomes between the DT group and the conventional ergometer group. However, the DT group demonstrated significant within-group improvements in the MMSE and TMT-J Part A. Exploratory analyses also suggested potentially favorable findings for selected cognitive outcomes and for mobility among participants with relatively preserved cognitive function. Thus, although seated DT ergometer training was not shown to be superior to conventional ergometer training, the findings suggest that it may have potential as a cognitive–motor training approach that warrants further investigation.

The unadjusted between-group effect sizes for the primary change-score comparisons were small (*r* = 0.065–0.143), and all adjusted 95% CIs included zero. The point estimates for DT walking time and TUG favored the DT group by approximately 1 s, but the corresponding CIs were compatible with a modest benefit, little or no benefit, or a slight disadvantage. Because clinically important thresholds were not prespecified and may not be directly transferable to this heterogeneous convalescent inpatient population, the clinical relevance of these estimates should be interpreted cautiously. Taken together, the findings do not demonstrate a clinically clear additional benefit of seated DT ergometer training over conventional ergometer exercise.

Walking times and TUG improved in both groups, but no significant between-group differences remained after adjustment for baseline values, age, and diagnosis. Similar findings were obtained in the sensitivity analysis restricted to participants scheduled to receive six training sessions per week, suggesting that differences in scheduled training frequency alone did not explain the results. The post hoc sensitivity analysis excluding episodes with changes in walking-aid use also yielded nonsignificant adjusted differences. Improvements in both groups may have reflected spontaneous recovery, conventional rehabilitation, repeated assessment, and the effects of ergometer exercise itself. Therefore, the observed improvements in mobility cannot be attributed specifically to the cognitive component of DT training.

Although the DT group showed improvements in the MMSE and TMT-J Part A, neither the between-group comparisons of change scores nor the adjusted analyses were statistically significant. Accordingly, the present findings do not demonstrate an additional cognitive benefit of DT training beyond cycling exercise alone. Nevertheless, in an exploratory sensitivity analysis restricted to participants who completed the TMT-J within the standard discontinuation limits, adjusted post-training Part A completion time was shorter in the DT group. Because TMT-J Part A primarily reflects visual scanning, attention, and processing speed [10], this finding may suggest that an association between seated DT training and these cognitive domains is more readily detectable among patients who can complete the assessment under standardized conditions. However, this analysis included a smaller and functionally selected sample and should therefore be interpreted as hypothesis-generating rather than confirmatory.

No clear improvement was observed in TMT-J Part B. Part B is more demanding than Part A and places greater emphasis on set shifting and cognitive flexibility [10]. In this study, paired Part B data were available for fewer participants, and missing data were particularly frequent in the conventional ergometer group. The findings for Part B may therefore have been influenced by both limited statistical power and selective inclusion of participants who were able to complete the more difficult task.

Studies have reported inconsistent findings regarding whether cognitive–motor DT training provides additional benefits beyond physical exercise alone [5,7]. Meta-analyses in older adults have reported improvements in global cognition, executive function, walking ability, and balance, although substantial heterogeneity has been noted across studies [4–6]. Cycling-based DT interventions have not consistently demonstrated superiority over aerobic exercise alone [7], whereas a randomized trial of seated DT resistance exercise reported an additional improvement in global cognitive function measured by the MMSE [13]. More recently, an umbrella review encompassing 17 reviews and 81 unique studies in older adults with neurocognitive disorders reported small meta-level improvements; however, physical outcome effects were nonsignificant in study-level analyses accounting for overlapping primary studies, and prediction intervals were nonsignificant for all outcomes [14]. The umbrella review highlights that average pooled effects may not translate consistently across settings and populations. Taken together, the available evidence suggests that the effects of DT training may depend on participant characteristics, exercise modality, cognitive task, training dose, comparator intervention, and selected outcome measures, and reinforces the need for cautious interpretation of the present exploratory findings.

In the exploratory subgroup analysis, participants with relatively preserved baseline MMSE scores showed greater improvements in DT walking time and TUG in the DT group. Patients with better-preserved cognitive function may have been better able to engage effectively with the simultaneous motor and cognitive demands of the training. However, the subgroup sample sizes were small, no adjustment was made for multiple comparisons, and the results may have been influenced by chance, baseline imbalance, or regression to the mean. Therefore, baseline MMSE should not currently be used to select candidates for seated DT ergometer training, although it may be a useful stratification factor in future prospective studies.

A potential clinical advantage of seated DT ergometer training is that it enables simultaneous lower-limb exercise and cognitive engagement with lower balance demands than walking-based DT training. During the early stages of convalescent rehabilitation, ambulatory DT practice may be limited by impaired balance, reduced walking ability, fatigue, or medical restrictions. Seated DT ergometer training may therefore provide a practical method of introducing cognitive–motor training before walking-based DT practice becomes feasible. However, it should be regarded as an adjunct rather than a substitute for task-specific gait training and subsequent ambulatory DT practice.

This study has some limitations. First, its retrospective and nonrandomized design precludes causal inference. Training modality was selected according to routine clinical and operational factors, including equipment availability, rehabilitation scheduling, and therapist judgment, and therefore selection bias and residual confounding cannot be excluded. Second, outcome assessors were not prospectively blinded, and it was not possible to determine whether they were aware of group allocation. Third, the actual number of completed sessions and cumulative training dose could not be determined; therefore, adherence and dose–response relationships could not be evaluated. Fourth, missing data may not have occurred at random. In particular, participants with poorer cognitive performance may have been less likely to complete TMT-J Part B. In addition, discontinuation procedures for the TMT-J were not consistently applied in routine clinical practice, and the primary analyses included some completion times exceeding the standard limits. For the mobility outcomes, it was not possible to retrospectively determine whether missing values reflected assessments that were not performed or results that were not entered into the dataset. Walking-aid use changed between assessment points in nine rehabilitation episodes and may have influenced mobility outcomes, although the changes occurred in both groups (four DT and five conventional ergometer episodes). A sensitivity analysis excluding these episodes produced findings consistent with the primary analyses, but residual heterogeneity in assessment conditions cannot be excluded. Fifth, no a priori sample size calculation was performed, and the nonsignificant between-group findings may have reflected limited statistical power. Sixth, no cycling-specific outcome, such as cadence, power, workload progression, or DT cycling performance, was available for analysis. Consequently, task-specific changes in ergometer performance may have been missed, and the extent to which any training effects transferred from seated cycling to gait-related outcomes could not be determined. Finally, the subgroup and TMT-J sensitivity analyses were exploratory, and the effects of cognitive and mobility changes on daily activities, falls, and participation were not evaluated.

## Conclusions

Seated DT ergometer training was implemented in routine convalescent rehabilitation and was accompanied by improvements in cognitive and mobility outcomes, but it did not demonstrate a clear advantage over conventional ergometer training. Exploratory findings suggested potential associations with visual attention and processing speed and with mobility among patients with relatively preserved cognitive function. Seated DT ergometer training may represent a cognitive–motor training option for patients who are not yet able to safely perform walking-based DT practice. Prospective randomized studies with a standardized training dose are required to determine its specific effects and identify the patients most likely to benefit.

## Supplementary Material

Supplemental Material

## Data Availability

The datasets generated and/or analyzed during the current study are not publicly available due to institutional and ethical restrictions but are available from the corresponding author on reasonable request.
