# Supplementary material for: Associations of seated dual-task ergometer training with cognitive and mobility outcomes in older adults with disabling conditions undergoing inpatient convalescent rehabilitation: a retrospective observational study
Source: Ann Med. 2026 Oct 3;58(1):2732560. doi: 10.1080/07853890.2026.2732560 (PMC13644430; doi:10.1080/07853890.2026.2732560)
Supplement: Supplementary file 1 [file IANN_A_2732560_SM7237.docx]

**Supplementary Material**

**Supplementary Table S1. Secondary cognitive and mobility outcomes**

| **Secondary outcome** | **DT group, n** | **DT group Δ (95% CI)** | **Conventional**  **ergometer group, n** | **Conventional ergometer group Δ (95% CI)** | **Between-group P** | **Effect size, r** |
| --- | --- | --- | --- | --- | --- | --- |
| TMT-J Part B (s) | 61 | −22.43 (−48.81 to 3.94) | 27 | −41.04 (−123.21 to 41.13) | 0.342 | 0.101 |
| ΔTMT (s) | 61 | −9.44 (−38.23 to 19.35) | 27 | −27.65 (−104.99 to 49.70) | 0.260 | 0.120 |
| 10-m comfortable walking time (s) | 78 | −3.04 (−4.34 to −1.74) | 46 | −2.47 (−3.69 to −1.25) | 0.548 | 0.054 |
| 10-m maximal walking time (s) | 78 | −2.89 (−3.77 to −2.00) | 45 | −1.96 (−2.90 to −1.02) | 0.680 | 0.037 |
| DT gait cost (%) | 77 | −8.12 (−17.07 to 0.83) | 40 | −0.75 (−8.37 to 6.88) | 0.413 | 0.076 |

Values are presented as mean changes from baseline with 95% confidence intervals. Negative values indicate shorter completion times or lower dual-task gait cost after training. Between-group comparisons of change scores were performed using the Mann–Whitney U test. Effect sizes were calculated as the absolute value of the standardized Z statistic divided by the square root of the total sample size for each comparison. ΔTMT was calculated as TMT-J Part B completion time minus Part A completion time and was analyzed only in participants with valid Part A and Part B values at both baseline and post-training assessments. Analyses were based on available cases, and missing values were not imputed.

Abbreviations: CI, confidence interval; DT, dual-task; TMT-J, Trail Making Test–Japanese; ΔTMT, Part B completion time minus Part A completion time.

**Supplementary Table S2. Sensitivity analyses of the primary outcomes according to scheduled training frequency**

| **Outcome** | **Analysis** | **n** | **Adjusted difference,**  **DT – conventional ergometer** | **95% CI** | **P** |
| --- | --- | --- | --- | --- | --- |
| MMSE | Primary analysis: all participants | 120 | 0.08 | −0.78 to 0.93 | 0.862 |
|  | Sensitivity analysis: 6 sessions/week | 99 | 0.24 | −0.67 to 1.15 | 0.607 |
| TMT-J Part A | Primary analysis: all participants | 115 | −6.59 | −22.06 to 8.87 | 0.403 |
|  | Sensitivity analysis: 6 sessions/week | 96 | −11.74 | −28.91 to 5.44 | 0.180 |
| DT walking time | Primary analysis: all participants | 117 | −0.89 | −2.77 to 0.99 | 0.354 |
|  | Sensitivity analysis: 6 sessions/week | 95 | −0.73 | −2.81 to 1.36 | 0.495 |
| TUG | Primary analysis: all participants | 118 | −0.97 | −2.62 to 0.67 | 0.246 |
|  | Sensitivity analysis: 6 sessions/week | 96 | −1.24 | −2.98 to 0.50 | 0.161 |

Adjusted differences represent estimated between-group differences in post-training values from ANCOVA models, with the DT group minus the conventional ergometer group reported. All models were adjusted for the corresponding baseline value, age, and diagnosis, with diagnosis entered as a categorical variable. Heteroscedasticity-consistent HC3 robust standard errors were used. The sensitivity analyses were restricted to observations from patients scheduled to receive six training sessions per week. Analyses were based on available cases, and missing values were not imputed.

Abbreviations: ANCOVA, analysis of covariance; CI, confidence interval; DT, dual-task; MMSE, Mini-Mental State Examination; TMT-J, Trail Making Test–Japanese; TUG, Timed Up and Go test.

**Supplementary Table S3. Exploratory sensitivity analyses of TMT-J outcomes restricted to standard discontinuation limits**

| **Outcome** | **DT group, n** | **DT group Δ (95% CI)** | **Conventional ergometer group, n** | **Conventional ergometer group Δ (95% CI)** | **Between-group P** | **Effect size, r** | **Adjusted difference, DT – conventional ergometer (95% CI)** | **Adjusted P** |
| --- | --- | --- | --- | --- | --- | --- | --- | --- |
| TMT-J Part A (s) | 48 | −15.29 (−22.89 to −7.70) | 28 | −6.45 (−21.66 to 8.75) | 0.182 | 0.153 | −15.86 (−30.64 to −1.09) | 0.035 |
| TMT-J Part B (s) | 46 | −14.09 (−29.19 to 1.00) | 20 | 1.98 (−9.86 to 13.83) | 0.127 | 0.188 | — | — |
| ΔTMT (s) | 38 | −3.20 (−22.52 to 16.12) | 16 | 8.33 (−12.76 to 29.42) | 0.280 | 0.147 | — | — |

Values are presented as mean changes from baseline with 95% CIs. Negative values indicate shorter completion times after training. Analyses were restricted to participants with recorded completion times within the standard TMT-J discontinuation limits of 180 s for Part A and 300 s for Part B at both baseline and post-training assessments. For ΔTMT, participants were included only when both Part A and Part B completion times were within the corresponding limits at both assessment points. Between-group P values were obtained using the Mann–Whitney U test. Effect sizes were calculated as the absolute value of the standardized Z statistic divided by the square root of the total sample size for each comparison.

For TMT-J Part A, the adjusted difference represents the estimated between-group difference in post-training values from ANCOVA, with the DT group minus the conventional ergometer group reported. The model was adjusted for baseline Part A completion time, age, and diagnosis, with diagnosis entered as a categorical variable, and used heteroscedasticity-consistent HC3 robust standard errors. Adjusted analyses were not performed for Part B or ΔTMT because they were not designated as primary outcomes. Analyses were based on available cases, and missing values were not imputed.

Abbreviations: ANCOVA, analysis of covariance; CI, confidence interval; DT, dual-task; TMT-J, Trail Making Test–Japanese; ΔTMT, Part B completion time minus Part A completion time.

**Supplementary Table S4. Sensitivity analyses of the primary mobility outcomes after excluding rehabilitation episodes with changes in walking-aid use**

| Outcome | Analysis | n | Adjusted difference, DT – conventional ergometer | 95% CI | P |
| --- | --- | --- | --- | --- | --- |
| DT walking time | Primary analysis: all participants | 117 | −0.89 | −2.77 to 0.99 | 0.354 |
|  | Sensitivity analysis: walking-aid use unchanged | 109 | −1.15 | −3.04 to 0.75 | 0.236 |
| TUG | Primary analysis: all participants | 118 | −0.97 | −2.62 to 0.67 | 0.246 |
|  | Sensitivity analysis: walking-aid use unchanged | 110 | −1.30 | −3.00 to 0.40 | 0.135 |

Adjusted differences represent estimated between-group differences in post-training values from ANCOVA models, with the DT group minus the conventional ergometer group reported. All models were adjusted for the corresponding baseline value, age, and diagnosis, with diagnosis entered as a categorical variable. Heteroscedasticity-consistent HC3 robust standard errors were used. The sensitivity analyses excluded the nine rehabilitation episodes in which walking-aid use changed between baseline and post-training assessments (four DT and five conventional ergometer episodes). Analyses were based on available cases, and missing values were not imputed.

Abbreviations: ANCOVA, analysis of covariance; CI, confidence interval; DT, dual-task; TUG, Timed Up and Go test.
